# The Case of Mr. Holder; with Some Cursory Remarks on the Existence of Polypose Concretions in the Heart
*See page 213.


**Published:** 1785

**Authors:** 


### LONDON MEDICAL JOURNAL,

FOR THE YEAR 1785,

### PART THE THIRD.

### SECTION I.

### ESSAYS AND OBSERVATIONS.

I. *The Case of Mr. Holder*; with some cursory Remarks on the Existence of polypose Concretions in the Heart. By Mr.* Richard Browne Ceston, F. R. S. *Consulting Surgeon to the General Infirmary at Glocester.*

MR. Holder, about forty years of age, of an athletic constitution and active disposition, towards the latter end of the year 1774 was frequently, when he walked fast, attacked with an uneasy sensation in his chest, particularly under the sternum, which, upon checking the degree of motion that brought it on, almost as instantaneously vanished.

* See page 213.

He firſt perceived this uneaſineſs about four years before, and obſerving it to come on chiefly when walking ſoon after a meal, and that the ſenſation was much like what he had felt upon ſwallowing haſtily a large draught of ſmall beer, which painfully diſtended the oeſophagus, he conſidered it as ariſing merely from flatulence; and thinking himſelf in every other reſpect in the higheſt degree of health, he paid little attention to theſe attacks, and was ſcarcely ever heard to ſpeak of them, unleſs to thoſe friends who were in company with him at the time they happened.

The high degree of reputation he had moſt deſervedly gained as a ſurgeon and apothecary, had thrown him into a very extenſive buſineſs, and of courſe he was continually expoſed to the uncertainty and inclemency of the weather, from which he ſuffered much at various times in the year 1774.

It may not be improper to obſerve here, that, to the age of twenty-one, he was one of the moſt temperate and healthy of men. In his twenty-ſecond year he was attacked with a jaundice, which continued ſix weeks, and was at laſt cured upon the diſcharge of a large gall ſtone. From that time he became ſubject to great great ſecretions of bile, which frequently paſ-ſed off both by vomit and ſtool, and were attended with ſuch exquiſite pain in the ſtomach and inteſtines as could only be allayed by opiates. Finding, by experience, that a ſtout, generous cyder was the beſt preſervative againſt theſe attacks, he made it his conſtant liquor, till its ſcarcity in the years 1772 and 1773 prevented his procuring it as uſual. From this time he took to beer.

About Auguſt, 1774, finding his ſtomach much diſordered, and the powers of digeſtion impaired, he left off malt liquors, and made uſe of cold water with toaſted bread in it, drinking but little wine after dinner, and in the evening a few glaſſes of rum and water. At firſt he imagined that he was much relieved by this alteration; his appetite was reſtored, and his food, which he ate rather voraciouſly, paſſed eaſily off his ſtomach. He, therefore, continued in this courſe for near three months, when, obſerving that he began to loſe fleſh, and feeling his ſtrength and ſpirits unequal to his buſineſs, he returned to his former way of living, and drank his wine as before.

In the month of January, 1775, the complaint in his cheſt became more frequent, and its paroxyfms feemed to increafe in degree as well as duration; but determining, if poffible, to be fuperior to it, he purfued his bufinefs with his ufual diligence, and feldom rode lefs than thirty or forty miles every day. At the end of this month he was firft attacked with the complaint while he was on horfeback; but, upon checking his pace, he found it paffed off as ufual when he was on foot. However, from this time he feldom rode without being more or lefs fenfible of it, till, by degrees, it became fo repeatedly troublefome, that he could not bear the motion of his horfe, if his pace exceeded a walk.

At the beginning of February, after being caught in a violent fhower of rain, he was feized with a cough, hoarfenefs, and fuch a proftration of ftrength, as confined him to his houfe. In this ftate I vifited him, and learned the following farther particulars: — That the uneafinefs he formerly ufed to feel upon motion, and which feldom attacked him when at reft, was, for the laft week, increafed to a conftant and fixed pain under the fternum, which, at that time, affected his refpiration fo much, that, to walk up ftairs, or even acrofs the room, unlefs in the moft gentle and equal pace, threatened immediate ſuffocation: nay, that he ſuffered nearly as much even upon attempting to get into bed, though he varied his poſition with the greateſt caution and care; but that after a time, when the effects of this fatigue were over, he frequently fell into a kind ſleep, and paſſed a refreſhing night. When he got up in the morning his pain returned; nor was he at eaſe till ſome time after he had ſettled himſelf in his ſitting room for the day. Of late his pain had extended itſelf farther into the right ſide of the thorax, and upwards to his neck, where he was conſtantly ſenſible of a noiſe, ſimilar to that of a *ſtream of water paſſing over obſtructions, or forcing a paſſage through a narrow, confined place*; but that upon ſome occaſions, when it became more remarkable, he would compare it *to the daſhing of water when ſtruck with a pole* *. His pulſe at the wriſt was in general hard and contracted, without the leaſt intermiſſion; but the motion of his heart could not be felt, though ſearched for with the greateſt attention. His hands and feet were always cold, and ſo benumbed, that

* The words in italics are to be conſidered as the particular expreſſions of Mr. Holder.

very little feeling remained in them: the lower parts of his legs were ſlightly oedematous.

Upon a ſuppoſition that the increaſe of his complaints aroſe from a recent cold, eſpecially as he now had a moſt troubleſome cough, an unuſual ſymptom in his former ſtate, he loſt about ſix ounces of blood, which afterwards ſeemed to have reduced him very much. This blood ſhewed little ſigns of inflammation, but the ſerum bore a very high bilious tinge. For the relief of theſe ſymptoms he preſcribed for himſelf, lac ammoniaci, oxymel ſcillitic. &c. and a bliſter on the ſternum.

His complaints, however, increaſed. The guſhing noiſe in the thorax ſeemed to himſelf ſo much more ſtrong and loud, that he often expreſſed his ſurpriſe at its not being heard by thoſe who were in company with him. The pain likewiſe became at times ſo great, that, though he bore it with the utmoſt reſolution, it was ſtrongly expreſſed by a great alteration in his countenance; and as his ſtrength daily diminiſhed, he found himſelf much more at eaſe by continuing almoſt conſtantly in an horizontal poſture. In this ſituation he was often ſo comfortable and free from any diſagreeable ſenſations, that he would converſe with as much cheerfulneſs and power as though not liable to ſuch dreadful attacks.

He now found that preſſure on the carotid artery quite ſilenced the noiſe in the thorax; but if it was continued for any time, or with any degree of force, he ſuffered an increaſe of pain, which was not entirely removed till the noiſe returned to its uſual ſtate. An opinion having been given that his complaints were occaſioned by an aneuryſmatical enlargement of the ſubclavian artery, the two carotids were frequently examined with conſiderable attention; and at one time it had been thought that the pulſation of the right was ſtronger than that of the left, but of this I was never ſenſible, though I endeavoured to aſcertain it with the greateſt care and exactneſs. His appetite, however, continued good, and he took the kind of diet that was thought fit for him with the higheſt reliſh, and moſt commonly found himſelf much refreſhed by it. As he was about this time more coſtive than was proper, clyſters were ſtrongly recommended to him as often as neceſſary; but a particular diſlike he had to this kind of aſſiſtance, induced him to have recourſe to another, the effects of which, as well as other ſubſequent circumſtances, are beſt deſcribed by himſelf in the following letter which I received from him in February, 1775:

"On Tueſday, February 21, I had a very

"tolerable day. Wedneſday morning, not

"being able to ſtrive for a ſtool, I attempted

"to put up a ſuppoſitory; I did effect it, but

"I had the moſt violent conflict between life

"and death I had yet experienced; however,

"I afterwards paſſed a good day and a good

"night. At ſix o'clock, Thurſday morning,

"after taking a little ſago, I vomited once,

"ſlept again, and at night took my pills.

"In half an hour after I had the moſt horrid

"bitter taſte in my mouth, and immediately

"ſuch a vomiting of very thick yellow bile,

"as I feared would have demoliſhed me. I

"drank warm water, and brought up a pint

"of clear bile: it then ruſhed violently down-

"wards, and I had the moſt highly bilious

"ſtool I ever ſaw. I drank chicken water,

"took clyſters of the ſame, and, in ſhort,

"waſhed the canal clean. About two o'clock

"I ſlept for an hour, then eat ſome broth and

"minced chicken, drank a little negus, and

"ſupped upon toaſt and new cyder, which,

"without exception, is the moſt delicious

"nectar.

" nectar. This, I think, may be called a bi-

" lious disease. I have no reason to believe

" it was by any means provoked. I passed

" the night well, and have been astonishingly

" better this day in every respect. One thing

" be pleased to observe, my heart was never

" once affected, and I have walked more and

" better this day than for a week past. I have

" resolved upon aloes succotrin. gr. v. calomel

" gr. ij. this evening, to prevent any more

" such lodgements, as there was much costive

" matter with the bile. Is it possible that bile

" should occasion such complaints as mine?

" (alluding to that in the thorax.) My gush-

" ing is very strong, and now got into my

" right ear. My wife and I are both sensible

" of the apex of my heart being felt in the

" common place."

Though he appeared better after this for a day or two, yet he found some slight returns of his bilious complaints, more particularly in the morning, and therefore he took *rad. colombo* gr. x. *spec. aromat.* gr. iij. every three hours, which relieved his stomach, and seemed to have such an effect on his intestines, that he passed, with tolerable ease, several black purging stools, somewhat like melted pitch, but not in the leaſt fœtid; he, therefore, continued theſe powders regularly, and always found them invigorate his ſtomach, raiſe his ſpirits, and revive the powers of nature.

He now obſerved that the remiſſion of his complaints, which uſed to be in the day time, took place in the night, and was attended with this circumſtance—that on the well night he could move in the bed, or even lie in any poſture, and as low as poſſible, with ſuch comfort, as to ſleep almoſt through the night; whereas, on the night of exacerbation, the motion of his heart was ſo languid, that his poſition could not be varied, even in the leaſt degree, without the utmoſt care and caution; for, unleſs this was attended to, *the circulation*—to uſe his own expreſſion—*became confuſed, and, as it were, wholly carried on in one corner of his heart, which, at ſuch times, beat with a whiz.*

On the 17th of March, upon a ſuppoſition that his complaints might ariſe from ſpaſm, he was recommended to try the effects of æther, by which the numbneſs in his legs and feet was ſurpriſingly relieved, indeed for a time removed, and every violent attack of pain in his cheſt inſtantly mitigated. On his firſt taking this medicine, which was in the quantity of two two tea-ſpoonfuls, he ſaid, he had a *ſenſation of ſuch a burſting within*, and particularly at the part where the pain was ſituated, that he was terrified for the conſequences; but his ſubſequent eaſe ſoon diſperſed his apprehenſions. From this time the pain, when it returned, ſeemed to have changed its ſeat, and, deſcending almoſt as low as the xiphoid cartilage, became more conſtant than before, though it often varied in degree.

As his ſtrength diminiſhed, the noiſe appeared to leſſen, and now and then almoſt ceaſed, at which times he ſuffered the greateſt anxiety and oppreſſion at his heart. Upon ſuch occaſions he had immediate recourſe to æther, which, invigorating the circulation, produced a return of the noiſe to its uſual, and ſometimes to an increaſed degree. He had, indeed, through his illneſs, felt the greateſt pain in his cheſt when the noiſe ſeemed leaſt; ſo that, when ſpeaking of his ſituation, he had a common expreſſion — *that Guſh was his friend; while Guſh ſtood by him he ſhould live.*

His countenance was now very ſallow, and he became ſenſible of a noiſe in the left ſide, ſimilar to, but much weaker than that in his right. At times he ſuffered a ſlight dyſpnœa, and frequently found it neceſſary, for greater eaſe, to change his horizontal poſition to one rather erect. The circulation grew daily more languid, and the powers of nature drooped apace.

With his ſtrength the noiſe in his cheſt gradually leſſened; ſo that, in the laſt week of his life, he found nothing of it in either ſide. About this time he frequently complained of a moſt violent pain in his head, and at intervals ſeemed to ſuffer ſuch an oppreſſion there, as brought on a ſtate of inſenſibility for many minutes. During this paroxyſm, he appeared, by his frequent ſighings and deep inſpirations, to undergo a ſlight ſuffocation. His pulſe was very ſmall and low, his extremities quite cold, ſo that he was often thought by thoſe about him to be in a dying ſtate: at length, after having appeared to doze a little, he awaked ſeemingly refreſhed, and would then ſay he was much better; upon which his pulſe grew ſtronger, his countenance more lively, and his extremities recovered their warmth proportional to the other parts of his body. In this manner he paſſed three or four days, viſibly ſinking under the repeated attacks, till March 31, when he died.

The

*The Diſſection.*

Upon laying open the abdomen, the omentum and inteſtines appeared remarkably loaded with fat, as did the peritoneum, but particularly ſo in the umbilical region, and around the *ligamentum ſuſpenſorium hepatis*. The liver was of its natural ſize and appearance, nor, upon being cut into, was there any deviation from its proper colour and texture. The gall bladder in no reſpect remarkable, and the bile in its uſual quantity. In ſhort, all the viſcera of this cavity bore the ſtrongeſt marks of health and vigour. Upon raiſing the ſternum with the greateſt caution, we found the mediaſtinum in a perfectly ſound ſtate, the cellular membrane retaining the moſt healthy appearance I ever met with, even in ſubjects who had ſuffered a violent death.

The external ſurface, or coat of the pericardium, was ſo covered with fat, that there was not the leaſt appearance of membrane to be ſeen. The internal was of its uſual appearance, unleſs exactly oppoſite the right auricle, where, for about the breadth of a ſhilling, it was beſet with ſome firm fleſhy granulations near the ſize of millet ſeeds. The liquor it contained was natural both in quantity and quality.

The heart *in ſitu* was longer and more pointed than uſual for its ſize. The right auricle much enlarged and very thin, bearing ſtrong marks of inflammation. The ventricle, having loſt its uſual firmneſs and colour, was ſo tranſparent, that I could, as it were, ſee into its internal ſubſtance. Upon cutting into theſe two cavities, a conſiderable quantity of air ruſhed out, and, upon laying them both open, they appeared as totally void of blood as if they had been waſhed clean. The interſtices between the *chordæ tendineæ* were full of air bubbles, much like thoſe on the ſurface of the blood when received in a porringer from an orifice in the arm. At the upper part of this ventricle, from between the *carneæ columnæ*, there aroſe a broad concretion *, of about the thickneſs of half a crown, of a light yellow colour, very denſe conſiſtence, and occupying near two thirds of the diameter of that cavity; it then roſe up into the auricle, and from thence was extended near four inches into the vena cava

* This concretion adhered ſo firmly to the carneæ columnæ, that it could not be ſeparated but by the ſciſſars.

ſuperior,

ſuperior, and about one inch into the inferior: its ſize in theſe veſſels was proportionate to their reſpective diameters. Another portion entered the pulmonary artery. The continuations or branches of theſe concretions were not ſo firm as the main body, and, in proportion as their diſtance increaſed, they aſſumed more and more the appearance of coagulable lymph, when a vein is opened, and diſcharges itſelf immediately into warm water.

The left ventricle was much loaded with fat, and, upon being ſlit up longitudinally to the extremity of its auricle, was found as void of blood as the right. From the carneæ columnæ of this cavity aroſe a ſmall concretion of a darker colour and more tender conſiſtence than that in the other cavity, paſſing into the aorta about an inch and half.

Both the ſemilunar and mitral valves were perfectly free from diſeaſe.

The lungs appeared in a very natural ſtate, unleſs on the right ſide, where they were firmly connected to the pleura, but with that kind of attachment which ſufficiently convinced us it was of long ſtanding.

*Remarks.*

Various were the conjectures on the nature of Mr. Holder's complaint, as well by thofe who vifited him during his illnefs, as by the moft eminent of the profeffion in London, whofe opinion he had himfelf taken by letter. It had, therefore, been fuppofed an aneuryfm of the fubclavian artery, difeafed valves of the heart*, angina pectoris, dropfy of the thorax, and, in fhort, every poffible diforder of that cavity.

From the time the pain became fixed under the fternum, he was himfelf fo poffeffed with the notion that matter was forming, or, at leaft, that his diforder was immediately feated in the mediaftinum,

* Being in London about three weeks before the death of Mr. Holder, I took that opportunity, and, at Mr. Holder's particular requeft, of talking over his cafe with Dr. Hunter, to whom I mentioned my fufpicions on the nature of his complaint, and the reafons which had led me to them. After an extenfive converfation on the topic of concretions, in which the Doctor acknowledged he had not yet met with fufficient reafons to determine abfolutely on the fubject, he faid, he could not help conjecturing that in this inftance the valves of the aorta were in a difeafed ftate, and either perforated, or fo circumftanced, as, by dividing the ftream of blood, to occafion mediaſtinum, that he took every opportunity, when I was with him, to ſtart a converſation on the propriety of perforating the ſternum, and as often declared his reſolution to undergo that operation, if I thought there was the leaſt probability of its being ſerviceable to him.

From two inſtances I had met with, where the peculiar ſymptom of a noiſe in the ear, like the daſhing of water, had been ſtrongly deſcribed by the patients, in whom I afterwards found a firm concretion in the right ventricle, I could not but mention my fears that the ſeat of his diſorder was wholly confined to his heart, and that he moſt probably had what was commonly called a polypus there. The notion, however, he had ſo ſtrongly taken up, and the

ſion the noiſe Mr. Holder was ſo ſenſible of. He then begged I would ſend him the reſult of the caſe, and appearances upon diſſection, as ſoon as I could collect them, which I did accordingly; but, without adding any remarks on the caſe, as I was not appriſed of the Doctor's intention to publiſh my letter till I ſaw it in the Medical Inquiries.

I muſt here obſerve, that the Doctor, at the time of our converſation, ſaid, that if concretions ſhould be met with in this caſe, the ſymptoms were ſo ſtrong and particular, that his doubts would in a great meaſure be cleared up. What were his ſentiments upon receiving the account I know not, as I never ſaw or heard from the Doctor afterwards.

doubts that the greateſt men of the profeſſion have ſo generally formed of the improbability of this circumſtance taking place during the life of the patient, put this opinion in an unſatisfactory light to him.

The idea of polypoſe concretions in the heart is to be found in early writers; but what with the philoſophy of the times, their erroneous phyſiology, and fondneſs for the marvellous, we have ſuch ſtrange accounts handed down to us, that no advantage could poſſibly be derived to practice from ſuch perplexing deſcriptions.

Morgagni, in his excellent book, *de Cauſis & Sedibus Morborum*, has collected together the ſentiments of moſt preceding authors on this ſubject, and, after making many valuable comments on them, very candidly concludes, that all the ſymptoms and obſervations then produced were too equivocal to lay any ſtreſs upon, ſince they were equally likely to ariſe from any diſturbed action of the heart and nerves, however excited.

What wonder then, if, from a want of a proper diagnoſtic, ſuch doubts ſhould have ariſen as to the probability of concretions exiſting during the life-time of the patient, that they

ſhould

ſhould have been almoſt baniſhed from the catalogue of diſeaſes.

From an attentive conſideration of facts alone, from a diſpaſſionate inquiry into appearances, ſupported and accounted for upon true phyſiological principles, muſt we draw that pathology, which will lead us, where we cannot cure, to diſtinguiſh properly, and perhaps relieve, as far as may be, ſome of the moſt diſtreſſing diſorders. I am well aware how cautious we ſhould be in drawing concluſions haſtily, or from ſingle caſes, and ſhall therefore, at preſent, only point out the circumſtances which particularly ſtruck me during my attendance on the unhappy ſufferer, and as illuſtrated by the appearances after death.

From the pulſe it has been ever ſuppoſed we muſt expect the ſtrongeſt indications of this complaint, and great dependence has therefore been placed on its intermiſſion as a ſure ſign of the exiſtence of ſuch concretions. This it will be of conſequence to examine into.

It has been related by many authors, that the moſt firm and denſe concretions are, in general, found in the right ventricle of the heart, and very frequently without the leaſt appearance of the ſame kind in the left. Conſidering

 this, this, then, as a fact, supported by undeniable authorities, the intermission of the pulse can by no means arise from any interruption this unusual substance in the right ventricle may appear to afford. For should it be so circumstanced as for the least space of time to block up the pulmonary artery, death must immediately ensue, through a surcharge from the quantity of blood perpetually returning to the heart; and if it should act in such a degree merely as to prevent the usual quantity from passing off as it ought to have done, it could only render the pulse smaller and quicker from the redoubled efforts of the heart to get rid of its unusual load. But as the pulse is well known to be regulated entirely by the propulsion of the blood from the left ventricle into the aorta, the concretion in the right ventricle cannot influence the action of that artery by means of the blood sent into the lungs; however, the action of these two cavities may be supposed in other respects to be affected by each other.

In complaints of the stomach and intestines we very frequently meet with an intermission of the pulse, which will entirely disappear as the affection in those parts is relieved. The pulse of a gentleman, who had a very obſtinate jaundice, frequently intermitted, and he was always ſenſible of this circumſtance when his ſtomach was particularly uneaſy. In another perſon, who had a cancer in the rectum, the pulſe uſually intermitted about every fifteenth ſtroke when the part was particularly painful to him. To this let me add a circumſtance which I met with in a patient who, when in perfect health, had always an intermitting pulſe, but in whom, when attacked with any feveriſh complaint, the pulſe, though much quickened, became perfectly regular in its ſtroke, and ſimilar in every reſpect to the uſual action of the artery under ſuch a feveriſh diſpoſition.

As this kind of pulſe is therefore an attendant on the diſeaſed ſtate of parts which have no other connection with the heart than by that general ſympathy ſo prevalent in the whole ſyſtem, and as in many inſtances where the pulſe has been remarkably intermitting, no particular morbid appearance could be obſerved in the heart, it can by no means deſerve to be conſidered as pathognomonic of any morbid ſtate of that viſcus, and is therefore equally to be excluded on this particular occaſion.

It

It may, however, be neceſſary to make a proper diſtinction between the irregular or unequal, and the intermitting pulſe; a circumſtance by no means ſo nicely attended to as it ought to be, and which probably firſt leading to the miſtake, might have introduced this affected action of the artery, as diagnoſtic of a diſorder with which it ſeems in no wiſe concerned.

While, then, we are under ſuch uncertainties on this ſubject, the preſent caſe marked with ſo many ſtrong and ſingular circumſtances, ſeems worthy our moſt ſerious attention; and though it may be ſaid that many of them appear in common with other affections of the thorax, we cannot but be ſtrongly impreſſed with that noiſe Mr. Holder ſeemed ſo ſenſible of, and the appearance of that firm concrete found in the right cavity of the heart, without the leaſt appearance whatever of bloody coagulum.

I before obſerved, that my having met with the noiſe here alluded to in two other patients, gave me the idea of it as a probable ſymptom of an exiſting concretion in this inſtance; yet, as in thoſe caſes there was ſome bloody coagulum connected with it, a doubt might have arifen arifen whether the feparation of the coagulable lymph did not take place, as has been imagined by many, *in articulo mortis.* But when we, in this inftance, reflect on the firmnefs of the concretion, as well as the total want of any bloody-coloured fubftance whatever, we muft confider it as totally diftinct from any probable feparation, as the powers of life declined, as well as that it muft have undergone the effects of repeated actions of the heart, to give fuch ftrength to its fubftance.

But a queftion may now occur, in what manner or from what caufe the concrete firft took its rife.

Mr. Hewfon has given us a cafe from Sir John Pringle's private notes, in which a cruft or polypus of the fhape and about the fize of a large pigeon's egg, divided lengthways, by ftrengthening the fide of the feptum, to which it adhered, prevented an abfcefs, containing about half a fpoonful of a dark reddifh-coloured matter of the confiftence of pus, from breaking into the left ventricle. This, he obferves, had probably been firft formed by a fecretion of lymph from inflamed veffels, and which, coagulating inftantly, was not wafhed off by the current of blood, as it would otherwise have been.

Here, then, we have an explanation well worth attending to, and drawn from a fair example of a concrete taking place from an inflammatory diſpoſition affecting one portion of the heart particularly.

To apply this inſtance to the preſent caſe, it will be proper to recollect, that though inflammation may ariſe from the many cauſes capable of increaſing the natural action of the heart when in a ſound ſtate, it may alſo be brought on from the increaſed efforts of this viſcus to perform its offices properly, when it has been weakened either by a general indiſpoſition of the whole body, or from the many cauſes which are known to affect it individually, ſuch as violent exerciſe, continued affections of the mind, &c. &c.

But, excluſive of this concrete originating from inflammatory exudation, we have reaſon to conclude that an analogy will hold good between it, and the formation of the coagulum generally found in aneuriſmal ſacs, when the coats of the artery have ſo far given way as to allow of a certain quantity of blood not to be duly duly acted upon in the dilated, and, of courfe, weakened portion of it.

Repeated inftances are upon record of the different degrees in which the right ventricle has not only loft the natural firmnefs of its texture, but has been enlarged into cavities of various unnatural fizes. Thefe are ftrong proofs of its diminifhed powers; and as the heart cannot, under fuch circumftances, act with fufficient energy to tranfmit onwards to the lungs the ufual quantity of blood received by the returning veins, a furcharge is likely to happen; and a feparation of fome of the denfer parts of the blood take place from its difpofition to coagulate when it has loft its proper progreffive motion. And will not the fafciculated ftructure of the ventricle be at all times ready to receive and retain any particle of blood difpofed and fit for feparation, and thereby lay the foundation of a future concrete?

In confidering how far the noife, Mr. Holder fo particularly defcribed, fhould be attended to as a diagnofis, we muft be very forcibly ftruck with the feveral circumftances attending it, the time and degree in which it had exifted, as well as the gradual advances it made up the neck and to the ear of the right fide; with the fenfe he had of it latterly on the left ſide, as he became leſs capable of moving himſelf; and conſequently compare them with the ſize and firmneſs of the concrete in the former, and its evidently more recent formation in the latter: to this if we add his feelings and deſcription, that, on the leaſt motion, *the circulation became confuſed*, and, *as it were, wholly carried on in a corner of his heart*, which, at ſuch times, *beat with a whiz* — that when the noiſe ſeemed leaſt, he felt the greateſt pain — and his remarkable expreſſion, that, *while Guſh ſtood by him, he ſhould live*, &c. we have the ſtrongeſt reaſon to infer, that the concretion was the ſource of theſe unhappy ſenſations, and that the foundation of it was very probably laid ſoon after the powers of the heart, and circulation through the lungs, were ſo evidently weakened. If we add to the above the noiſe ſo remarkable in the aneuriſmal varix, as well as that deſcribed by patients labouring under arterial dilatations, in which coagula are in general met with, we have additional confirmations of the effects which may be produced when the ſtream of blood is divided, or unequally acted upon by the ſides of its containing cavity.

 Thus

Thus far I have ventured to call forth the attention of practitioners to future obſervations on this ſubject. I ſhall at preſent only add my wiſh that the circumſtances now produced may rouſe up farther inquiries into a complaint of no ſmall conſequence, and which I am ſtrongly perſuaded obſervers will hereafter not only fully identify, but give a ſatisfactory explanation of.

Diſorders of the heart are numerous, and ſhould be much more clearly diſtinguiſhed than they are at preſent. How far the viſcera in general may be affected by an impaired function of this grand regulator, on which the whole powers of the circulation ſo immediately depend, muſt be obvious to every one.

Diſſections have afforded me ample proofs that more ſudden deaths are to be attributed to the heart than to the head; and, to ſpeak of one in particular, from the different reports given by thoſe gentlemen who have examined the thorax of ſuch as have ſunk under what is commonly called the angina pectoris, I think it would not be difficult to ſhow, that what has been attributed to ſo many cauſes may be very readily accounted for from a reduced action or weakened powers of the heart alone. For theſe opinions I ſhall offer my reaſons at ſome future opportunity.

